# Toxinology in the proteomics era: a review on arachnid venom proteomics

**DOI:** 10.1590/1678-9199-JVATITD-2021-0034

**Published:** 2022-02-28

**Authors:** Filipi Calbaizer Marchi, Edneia Mendes-Silva, Lucas Rodrigues-Ribeiro, Lucas Gabriel Bolais-Ramos, Thiago Verano-Braga

**Affiliations:** 1 Department of Physiology and Biophysics, Institute of Biological Sciences, Federal University of Minas Gerais (UFMG), Belo Horizonte, MG, Brazil.

**Keywords:** Proteomics, Scorpions, Spiders, Venomics

## Abstract

The word venomics was coined to acknowledge the studies that use omics to investigate venom proteins and peptides. Venomics has evolved considerably over the last 20 years. The first works on scorpion or spider venomics were published in the early 2000’s. Such studies relied on peptide mass fingerprinting (PMF) to characterize venom complexity. After the introduction of new mass spectrometers with higher resolution, sensitivity and mass accuracy, and the next-generation nucleotide sequencing, the complexity of data reported in research on scorpion and spider venomics increased exponentially, which allowed more comprehensive studies. In the present review article, we covered key publications on scorpion venomics and spider venomics, presenting historical grounds and implemented technologies over the last years. The literature presented in this review was selected after searching the PubMed database using the terms “(scorpion venom) AND (proteome)” for scorpion venomics, and “(spider venom) AND (proteome)” for publications on spider venomics. We presented the key aspects related to proteomics in the covered papers including, but not restricted to, the employed proteomic strategy (i.e., PMF, two-dimensional gel electrophoresis, shotgun/bottom-up and/or top-down/peptidome), and the type of mass spectrometer used. Some conclusions can be drawn from the present study. For example, the scorpion genus *Tityus* is the most studied concerning venomics, followed by *Centruroides*; whereas for spiders the studied genera were found more equally distributed. Another interesting conclusion is the lack of high throughput studies on post-translational modifications (PTMs) of scorpion and spider proteins. In our opinion, PTMs should be more studied as they can modulate the activity of scorpion and spider toxins.

## Background

Venomous animals have one or more venom glands, and they usually have a specialized apparatus to inject the venom into their prey or use it for defense. There are examples of marine and terrestrial venomous animals, such as *Physalia* sp*.* (phylum Coelenterata) and *Tityus* sp. (phylum Arthropoda), respectively. 

Scorpions and spiders (phylum Arthropoda, subphylum Chelicerate, class Arachnida) have their bodies divided into cephalothorax and abdomen. Scorpions have their venom apparatus located at the last abdomen segment named telson. Scorpions’ diet is based on arthropods and small animals, such as gecko (phylum Chordata, class Reptilia). There are 2,200 known scorpion species distributed over 19 families but the most studied one is the Buthidae (Koch, 1837), as it accounts for 95% of all reported scorpion accidents [1-4].

Buthidae scorpions are separated into two geographical groups. The Old-World scorpions are found mainly in Northern Africa, Southern Europe and the Middle East, while the New-World scorpions are distributed in the Americas [5]. Regarding scorpions of medical importance, the Old-World genera are represented by *Androctonus* (Ehrenberg, 1828), *Leiurus* (Ehrenberg, 1828), and *Buthus* (Leach, 1815), among others. The New-World genera include mainly *Centruroides* (Marx, 1890) and *Tityus* (Koch, 1836). Specifically, *Androctonus* sp., *Leiurus* sp. and *Buthus* sp. are distributed in the Saharo-Sindian zone, a vast and arid region spanning from northwestern Africa to India; whereas *Tityus* sp. are mainly distributed in South America and *Centruroides* sp. are found in Central America and Mexico [3]. 

In general, scorpion venoms are composed of salts, small molecules, peptides and proteins [e.g., bradykinin-potentiating peptides (BPPs), Nav-neurotoxins (NaTxs), Kv-neurotoxins (KTxs), Cav-neurotoxins (CaTxs), among others] [4-7]. Envenomation by *Androctonus* sp. are painful and can lead to cardiovascular manifestation as cardiac arrhythmia and ultimately heart failure [4]. *Leiurus* sp. sting cause local pain, burn and swelling. Systemic manifestations may include cardiovascular impairment (e.g., tachycardia, hypertension or hypotension, etc.), priapism, and vomiting [4]. 

The *Buthus* genus was subjected to a major taxonomical update, which reclassified as new species animals that used to be known as *B. occitamus*. Most epidemiological reports have been based on *B. occitamus* cases in Morocco and based on the current taxonomical classification this species is not found in this country. Therefore, more epidemiological data are required to state the common medical manifestations of accidents with this genus [4,8]. 

Regarding New-World genera, most common clinical manifestations of *Tityus* sp. accidents include local pain and burning at sting site. Systemic symptoms may include headache, vomiting, sweating, dizziness, hypersalivation, circulatory failure, cardiac arrythmias and respiratory arrest [4]. Regarding *Centruroides* sp. stings, the most frequent clinical outcomes include pain, local edema and fever. It can also lead to cardiovascular and respiratory impairments [4]. 

According to the “World Spider Catalog” (https://wsc.nmbe.ch/), there were 49,173 registered spider species by February 25^th^ 2021 distributed worldwide. Similar to scorpions, spiders use their venom for predation and defense. These arachnids have a pair of venom glands in each chelicera at the cephalothorax. Spiders’ diet is based on insects [9] but larger spiders, as *Lasiodora* sp. (Koch, 1850), can eat small vertebrates as well. 

Spider venoms may contain ions; small molecules, such as nucleotides, amines, amino acids, and polyamines; proteins (e.g., phospholipases, metalloproteases); and peptides (e.g., neurotoxins and insecticidal peptides) [10,11]. Accidents with spiders are registered in many countries but, interestingly, only four genera account for most severe accidents; *Atrax* (Pickard-Cambridge, 1877), *Loxosceles* (Heineken & Lowe, 1832)*, Latrodectus* (Walckenaer, 1805), and *Phoneutria* (Perty, 1833) [12,13]. *Loxosceles* sp. (family Sicariidae) and *Latrodectus* sp. (family Theridiidae) are distributed worldwide, whereas *Phoneutria* sp. (family Ctenidae) is mainly found in Central and South America, and *Atrax* sp. (family Atracidae) in Australia. 

*Loxosceles* sp. venom is mainly composed of phospholipases, metalloproteases, hyaluronidases, insecticidal peptides, among others [14]. Accidents can cause local necrosis and hemodynamic alterations, eventually leading to acute renal failure [15,16]. *Latrodectus* sp. venoms are composed of neurotoxic peptides for vertebrates (e.g., α-LTX), crustaceans (e.g., α-LCT) and insects (e.g., α-LIT) [17]. Widow spider (*Latrodectus* sp.) bites are painful and may lead to systemic manifestations, including nausea, headache, fatigue and ultimately injuries in the cardiac tissue [18]. *Phoneutria* has an aggressive behavior thus, accidents are not rare. A number of neurotoxins (~40 molecules as reported by [19]) from *P. nigriventer* venom has already been identified [19,20]. *Phoneutria* sp. envenomation causes local pain. It can also cause priapism and systemic envenomation, though it rarely leads to death [21,22]. *Atrax robustus*, the Sydney funnel-web spider, is a notorious species from the *Atrax* genus as it is acknowledged as the deadliest spider worldwide [18], mainly due to its Nav neurotoxins named δ-hexatoxins (δ-HXTXs) [23]. *Atrax* envenomation is painful and may lead to severe systemic and life-threatening effects, related with autonomic and neuromuscular excitation [18].

Even though spiders and scorpions cause health problems, there is a brighter side associated with them as scientists learnt over time that venom peptides are also associated with beneficial outcomes. Examples are: the anti-hypertensive peptide family in *Tityus* sp. venoms, named hypotensins [24-26]; a cryptic peptide from the hypotensin I (Ts14) is a potential cardioprotective agent [27]; the PnTx2-6 toxin from *Phoneutria nigriventer* venom that causes priapism [28] was latter redesigned in laboratory as a non-toxic peptide with potential application as an erectile dysfunction treatment [29]; anti-thrombotic and anti-inflammatory peptides [30,31], antimicrobial peptides [32,33], and bio-insecticides [34], etc.

The molecular diversity of scorpion and spider venoms are frequently acknowledged as “treasure chests” [35]. Several approaches are used to access the molecular diversity hidden inside venoms but the most popular one is probably the mass spectrometry-based proteomics. In this review we aimed at presenting key publications in venom proteomics, often referred to as venomics, in the context of scorpions and spiders, as well as recent advances in the field. We will also present recent advances in bioinformatics and proteomics that can assist in studying the proteome of scorpion and spider venoms. 

## Proteomics and its use in toxinology

Venom composition of many arachnid species has remained undefined for a long time due to limitations of traditional biochemical approaches to analyze small amounts of venoms that are usually extracted from spiders and scorpions [36]. Early studies to characterize scorpion venom components consisted in extensive chromatographic steps to isolate them and subsequent evaluation of their biological activity and potential three-dimensional structures [37], in a “function-to-structure” approach. However, progress in the omics field (genomics, transcriptomics and proteomics) allowed high throughput characterization of venom composition, and discovery of new peptides and proteins [38], in a “structure-to-function” approach.

Venomics has emerged by the use of proteomics to study venom composition. It can also refer to a broader omics (proteome, genome, transcriptome, metabolome) venom characterization [39], but in this review we will focus on venom proteomics. Although one of the firsts high throughput studies of an arachnid venom proteome was reported in the early 2000’s [40], mass spectrometry (MS)-based analysis was first used in 1979 to characterize venom metabolites from the Sydney funnel-web spider Atrax robustus by gas chromatography-mass spectrometry (GCMS) [41]. However, it was more difficult to use MS to study peptides and proteins before the 1980’s due to the lack of soft ionization techniques [42]. This problem was solved when the electrospray ionization (ESI) was invented by John Bennett Fenn in 1984 [43] and the matrix-assisted laser desorption/ionization (MALDI) was invented by Franz Hillenkamp and Michael Karas in 1985 [44]. 

**Figure 1.**
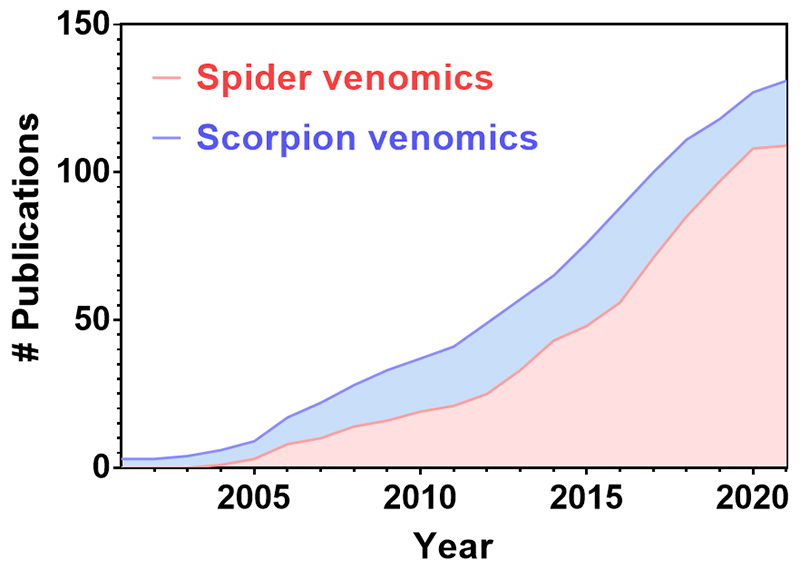
Number of publications on scorpion venomics and spider venomics. Results were retrieved from PubMed (https://pubmed.ncbi.nlm.nih.gov/) by March 2021 using the following search parameters: “(scorpion venom) AND (proteome)” for scorpion venomics or “(spider venom) AND (proteome)” for spider venomics.

Venomics has evolved substantially over the last 20 years. It is frequently employed to study arachnid venoms these days as seen by the exponential increment of publications using venomics (Figure 1). However, there are limitations on its use to study arachnid venoms as, not rarely, the genome or venom gland transcriptome of a given specie has not been sequenced. A way to circumvent this problem is by sequencing *de novo* venom peptides, either by manual interpretation of MS/MS spectra or assisted by algorithms that allow high throughput *de novo* peptide sequencing [45]. On this regard, Gorshkov et al. [46] published an algorithm to assist peptide *de novo* sequencing. 

**Figure 2.**
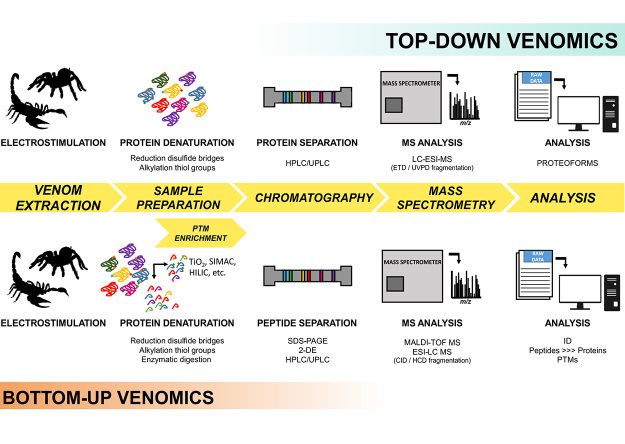
Venomic workflow. General workflows for top-down and bottom-up venomics are presented.

Different proteomic approaches allow characterizing venom compositions. There are many venomic workflows that can be employed but general workflows for top-down and shotgun/bottom-up are presented in Figure 2. Since arachnid proteomes are also subjected to post-translational modifications (PTMs), we included PTMs enrichment steps in the provided workflow (Figure 2) as we believe PTMs should be studied more in arachnid venoms. Importantly, detail protocols to study PTMs in general have been published [47,48] and can be employed in the venomic context as well.

## Bioinformatics in the context of arachnid venomic studies

UniProt, NCBI Genbank/GenPept, and the Protein Data Bank (PDB) provide large datasets, playing essential roles in providing access to information regarding protein sequences, three-dimensional structures (if available) and biological activity.

Throughout the years, attempts have been made to create new databases with more specific information on venom proteins and toxins. The International Venom and Toxin Database, the Tox-Prot program, the snake neurotoxin database, the scorpion toxin database, and the Animal Toxin Database (ATDB) were created to supply an early need to merge information about venom proteins [49]. However, most of them are based on unformatted text, restricted taxonomic groups, and lack of system effectiveness for data mining, resulting in discontinuing of the service or being incorporated by other databases [39,49]. On the other side, within UniProt, for example, the UniProtKB/Swiss-Prot Tox-Prot program, based on the Tox-Prot program, can provide access to venom protein sequences and functions from several venomous species [50]. The animal toxin annotation project, using the Tox-Prot program, aims at systematically annotate proteins secreted in animal venom, including spiders and scorpions, among other species [50,51]. In this respect, the Swiss Institute of Bioinformatics (SIB) developed a free web-resource regrouping information from the UniProtKB/Swiss-Prot database (manually annotated and reviewed) and UniProtKB/Trembl (automatically annotated) on venom proteins, mostly with toxic activity. Information access is divided into taxonomy, activity, venom protein families, and PTMs in venom proteins, having data about six taxa, including scorpions and spiders (https://venomzone.expasy.org/).

Although these databases are critical for comparison of toxins across different groups of venomous animals, there is no established standard for practical annotation of information about peptides and proteins from many venom species, especially the names of toxins, the description of the function, and the classification of toxins [51,52]. This lack of consistency leads to numerous duplications of entries and low efficiency for searches. This results in a barrier against data exchange and comparison, making data mining difficult and estimations imprecise. A few attempts on standardization protocols propose the use of machine learning-based classifiers. ToxClassifier is a machine learning web-based tool for the prediction of likely animal toxin sequences, allowing to distinguish toxins from non-toxin sequences. It also increases curation of existing databases by reporting the best-hit annotation and classifying a toxin into the most correct toxin protein family (http://bioserv7.bioinfo.pbf.hr/ToxClassifier/index.jsp; [53]). In contrast, specialized databases from venomous animals are slowly emerging. These databases are usually a rich information pool of manually curated content that deal with specific subsets of animal toxins [52].

SCORPION, launched in 2002, was a specialized database of scorpion toxins. Its main focus was to facilitate the design of experimental protocols [54]. The structure was designed to provide a basis for extending and clarifying the existing structural and functional classification of scorpion toxins data with easy integration of bioinformatics tools for additional analyses, like identification of sequence patterns associated with specific structural or functional properties of scorpion toxins [54]. An update, SCORPION2, with an increase in the records present in the database was launched a few years later. Combining search algorithms with prediction tools allowed users to extract and perform specific queries: text searches of scorpion toxin records, sequence similarity search, extraction of sequences, visualization of scorpion toxin structures, analysis of toxic activity, and functional annotation of previously uncharacterized scorpion toxins [55].

Another specialized database for scorpion toxins is the Kalium (http://kaliumdb.org/). This database is an open-access resource that collects manually curated data on potassium channel toxins (KTxs) purified from scorpion venom and provides an easy link to general databases such as UniProt, PDB, NCBI Taxonomy Browser, and PubMed.

On the other side, Arachnoserver (http://www.arachnoserver.org/) provided information on venoms from spider species. This manually curated database was centered on mature active peptides, containing 1,576 molecules as of October 2020, which were retrieved from UniProtKB. It contained information on the sequence, three-dimensional structure, and biological activity of protein toxins. All mature toxins in ArachnoServer were named according to the standard nomenclature for spider toxins proposed by King in 2008 [56], with the inclusion of alternative names found in the literature to facilitate researches. Its strategy focused on displaying one toxin sequence per entry on a page, providing cross-references to several databases, including the EMBL nucleotide data bank, which allows retrieval of the original nucleotide sequence submission.

**Figure 3.**
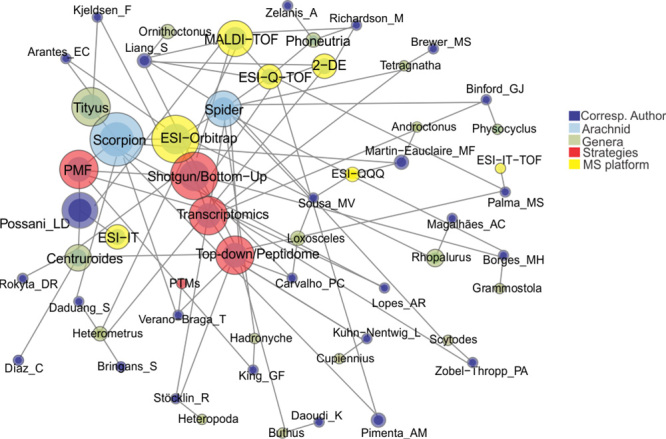
Summary of publications on venomics. Interaction map reporting the main terms (arachnid genera, venomic method, mass spectrometry platform, and corresponding author) found in the publications represented in this review. Node size is associated with the frequency that each term appeared in all publications.

In the next couple of sections, we will cite most publications on spider venomics and scorpion venomics. We will also provide a table with key information about the cited publications, as well as an interaction map that connects predefined “terms” (spider or scorpion, genera, use of transcriptomics, proteomic methods, MS platforms, and corresponding author) extracted from the publications (Figure 3).

## Spider venomics

One of the first studies on spider venomics was published in 2005. Machado et al. [57] used a range of proteomic techniques to access the venom proteome of the brown spiders (*Loxosceles gaucho, L. intermedia and L. laeta*; family Sicariidae), with particular interest in analyzing the dermonecrotic toxin loxnecrogin and its potential proteoforms. Two-dimensional gel electrophoresis (2-DE) showed that the potential loxnecrogin’ proteoforms spanned from *p*I ~ 4.4 to 7.3 and 30-35 kDa mass range. Gel bands corresponding with the potential loxnecrogin’ proteoforms were in-gel digested and subjected to peptide mass fingerprinting (PMF) by means of MALDI-TOF MS, and *de novo* sequenced by Edman degradation and MS/MS (ESI-Q-TOF MS). The authors hypothesized that toxins proteoforms (isoforms) might be related to evolutionary adaptation, maximizing both hunting and defense capabilities. Moreover, they emphasized how the purification and characterization of toxins in venom proteomes are fundamental to understand the physiopathology of envenomation.

Similarly, Richardson et al. [35] compared the partial proteome of spider venoms from the genus *Phoneutria* (*Phoneutria nigriventer*, *P. reidyi* and *P. keyserlingi*; family Ctenidae) in 2006. Out of 400 protein and peptide species detected in this study, 100 complete or partial sequences were obtained by Edman degradation and MS/MS (ESI-Q-TOF MS). Two new families of small toxins, some larger protein components, and two serine proteinases from the *P. nigriventer* venom were described. The authors also compared the *P. keyserlingi* venom proteome from male and female specimens by 2-DE, reporting a sexual dimorphism. 

Yuan et al. [58] reported a venom proteomic and peptidomic study of the Chinese “bird spider” *Ornithoctonus huwena* (family Theraphosidae). The authors employed gel filtration chromatography to separate peptides (MW < 10 kDa) from proteins (MW > 10 kDa). Venom proteins were separated by 1-DE and 2-DE. After in-gel digestion, proteolytic peptides were analyzed by ESI-Q-TOF MS/MS or MALDI-TOF-TOF MS. Protein identification was done by Mascot search engine. Separation of venom peptides (peptidome) was done by CIEX-HPLC followed by RP-HPLC. Peptide sequencing was achieved by MALDI-TOF MS and Edman degradation. 90 proteins (MW > 10kDa) were identified using the proteomic approach, including enzymes, binding proteins, and others. Using the peptidomic approach, the authors reported more than 100 components (MW < 10 kDa) in the *O. huwena* venom, including 47 sequenced peptides. Their findings showed pieces of evidence suggesting gene duplication, focal hypermutation and post-translational modifications (PTMs) in spider toxins as probable origin for the diversity of spider venom proteins and peptides.

Analytical methods evoked in a rapid pace over the last 20 years, including the introduction of the orbitrap mass analyzer [59] and next-generation mRNA and DNA sequencing. Venomics surfed on this innovative wave, boosting the complexity and depth of venom profiling, as seen by more recent publications on spider venomics [58-73], some of which shall be described in next paragraphs.

The study of Oldrati et al. [60] illustrates a rapid and efficient method for the analysis of venom composition based on venom glands mRNA sequencing and venom proteome profiling. Their focus was the analysis of cysteine rich peptide toxins from four different spider species: *Heteropoda davidbowie* (family Sparassidae), *Poecilotheria formosa* (family Theraphosidae), *Viridasius fasciatus* (family Viridasiidae) and *Latrodectus mactans* (family Theridiidae). This approach led to the profiling of 284 characterized cysteine rich peptides with high resolution, 111 of which were part of the Inhibitor Cysteine Knot (ICK) structural motif. The *H. davidbowie* venom revealed high diversity in venom composition, 32 peptides (of 95 identified peptide) were classified in 6 distinct families containing the ICK structural motif. The *P. formosa* venom accounted for 126 peptide sequences, with 52 ICK toxins being part of 3 distinct families. *V. fasciatus* venom contained 49 peptide sequences, with 22 ICK structural motif peptides from 5 families. The venom of *L. mactans* had 14 cysteine rich peptides, with 5 ICK toxins from 1 family (CSTX superfamily).

The work of Kuhn-Nentwig et al. [61] is quite interesting. Aiming to add new insights into the structure and function of spider venom toxins and their influence on the homeostasis of prey and/or aggressors, a comprehensive analysis of the venom gland transcriptome and proteome from *Cupiennius salei* (family Trechaleidae) was employed. The venom proteome of *C. salei* was studied combining bottom-up and top-down proteomics using LC-ESI-Orbitrap MS. Protein and peptide identification was performed using the UniprotKB database supplemented with sequences translated from the venom gland transcriptome. The authors detected 81 transcripts of neurotoxins from 13 peptide families, including 54 putative (based on transcriptome) neurotoxins. Their proteome approach allowed to validate the presence of 49 proteins out these putative 54 neurotoxins. Finally, the authors proposed a venom dual-mode of action, in which neurotoxins disable the prey or aggressor while metabolites impair animals’ homeostasis.

Diniz et al. [62] were interested in providing a broad screening of the venom proteins produced in the *Phoneutria nigriventer* venom glands. To accomplish this goal, they combined conventional and next-generation cDNA sequencing with Multidimensional Protein Identification Technology (MudPIT). Transcriptomic and proteomic data showed that cysteine-rich peptide toxins were the most abundant component in the venom. They also reported several potential variants or proteoforms of already described cysteine-rich peptide toxins, and novel ones with unknown function were identified too. The authors concluded that the observed relative abundance of insecticide toxins may have an important role in the envenomation of natural prey. 

Santana et al. [63] performed proteomics characterization of ontogenetic variation within a population of *Phlogius crassipes* (Australian tarantula) to investigate how spider venom composition may be influenced by different predatory niche factors such as sex, diet, habitat, and climate. This study revealed that *P. crassipes* venom changes continuously according to spider size, which could be due to a change in the preys that the spiders encounter at different life stages, mainly due to mating searches, as adult male specimens may incorporate toxins at this life stage that enable them to defend themselves from predators.

Sanggaard et al. [65] used comparative genomics as well as venomics to study the venom and silk proteomes from the African social velvet spider, *Stegodyphus mimosarum*, and the Brazilian white-knee tarantula, *Acanthoscurria geniculata*. The analysis of spider venom showed that both spider species contained a large repertoire of cysteine-rich peptides, which most likely mediate the toxic effects of the venom, possibly by processing and activating protoxins. They also found that the dragline silk of the velvet spider is composed by at least two types of spidroins (spider silk proteins). Four novel spidroin-related sequences were identified.

Tang et al. [67] used high throughput peptide identification techniques on the venom of the tarantula *Haplopelma hainanum* (*Ornithoctonus hainana*; family Theraphosidae), a highly venomous spider found in southern region of China. The authors employed three different approaches: i) transcriptomics, ii) peptidomics, and iii) genomics. Around 420 peptide toxins were detected by MS, and 272 peptide precursors were deduced from cDNA and genomic DNA sequences. After data processing, 192 mature sequences were identified by combining the three omics approaches. Peptide toxins could be classified into 11 superfamilies based on sequence similarity. Additionally, the results suggested a possibly gene duplication and focal hypermutation that could be responsible for the huge molecular diversity observed in spider peptide toxins.

One of the latest reports on spider venomics was published last year in PNAS. The authors employed several omics approaches to study what they called the “structural venomics” of the Australian funnel-web spider *Hadronyche infensa* (family Atracidae). For venom profile, they used a combination of LC-MS platforms to analyze intact peptides (peptidome) and tryptic digested peptides using the lab-made *H. infensa* venom gland transcriptome for database search (bottom-up proteome approach). Structural determination was done using NMR after expression of identified proteins. The authors detected 3,051 unique peptides in the venom of *H. infensa*. Based on this impressive identification number, they concluded that the *H. infensa* venom peptidome is one of the most complex in terrestrial venomous animals. Their proteome approach allowed the identification of 1,108 venom proteins out of the 1,224 predicted ones by the transcriptome approach. Finally, this work unveiled that the inhibitor cystine knot (ICK) toxins are highly dominant protein structures in the *H. infensa* venom [68].

## Scorpion venomics

One of the first scorpion venom proteome studies was performed in 2001. Pimenta et al. [40] performed a PMF of the *Tityus serrulatus* (family Buthidae) venom. The authors employed two MS platforms (online LC-ESI-QQQ MS and offline LC-MALDI-TOF MS) to analyze the toxic fractions of *T. serrulatus* venom, obtained by Sephadex G50 size exclusion chromatography, reporting over 300 ion species as potential venom toxins.

In 2004, Batista et al. [74] reported that proline-rich peptides from the *Tityus cambridgei* venom were prone to in-source fragmentation in ESI. The authors also characterized a new Nav toxin by Edman degradation and MS. In the following years, the New-World scorpions were subject to various venomic studies, including an interesting report of individual variability in *T. serrulatus* venom. Pimenta et al. [75] reported intra-specimen variation in the composition of *T. serrulatus* venom depending on starvation duration. Of course, inter-specimens’ variability was also observed [75]. Due to the importance of molecular phenotypes to the understanding of phylogenetic and ecological relations, many other proteomic studies analyzed intraspecific variations in scorpion venom proteins and peptides [76], including sexual dimorphism [77].

In 2006, Nascimento et al. [78] explored more the potential correlation of venom composition and interspecific variations. The authors evaluated whether venomics could be used to assist scorpions’ taxonomical classification in Buthidae family. Three species from the New-World (*Tityus stigmurus*, *T. serrulatus*, *T. bahiensis*) and two subspecies from the Old-World (*Leiurus quinquestriatus quinquestriatus* and *L. quinquestriatus hebraeus*) were used in this study. The authors used 2D-LC (CIEX and RP) and ESI-Q-TOF MS analyses to profile scorpions’ PMF. A phenetic correlation tree was provided based on venom PMF from each specie. Correlation was in agreement with the classical classification, showing that indeed venomic-based approaches may be used for taxonomical classifications.

Among the Buthidae scorpions, the genus *Tityus* is frequently studied by means of venomics [7,46,79-86]. Recently, a venomic study of *Rhopalurus agamemnon* New-World scorpion was reported [87]. *R. agamemnon* is a large scorpion (~11 cm) found in the Brazilian savanna, known as “Cerrado”. The authors performed a comprehensive characterization of *R. agamemnon* venom by bottom-up proteomics and enzymatic activity assays. The *Centruroides* is another New-World genus frequently subjected to venomic studies [88-93]. As observed in *T. serrulatus* venom by Pimenta et al. [75], it was reported individual variability in the Mexican scorpion *Centruroides limpidus* venom. Comparing the venom from male and female by PMF, and 2-DE followed by in-gel digestion and LC-ESI-Orbitrap MS analysis, the authors reported sexual dimorphism in *C. limpidus* venom [92]. Sexual dimorphism was also observed in *C. hentzi* venom [91]. 

Diaz et al. [89] studied the venom of *Centruroides edwardsii* by transcriptomics, proteomics, and bioassays. Venom proteomic analysis indicated the presence of a hyaluronidase, several cysteine-rich secretory proteins, metalloproteinases, and a peptidyl glycine α-hydroxylating monooxygenase like-enzyme. They also identified peptides similar to the Kv neurotoxin margatoxin, a dominant toxin in the venom of its related scorpion *C. margaritatus*. They also identified Nav-modulating peptides similar to other scorpion species from *Centruroides* and *Tityus* genera. 

Romero-Gutiérrez et al. [94] used transcriptomic and proteomic analyses to identify the components from the *Serradigitus gertschi* venom. They reported 119 annotated transcripts. The proteomic analysis revealed that 24 of the encoded peptides were indeed found in the venom. The study also revealed several unannotated transcript-derived peptides, demonstrating that there is still a number of scorpion venom components of unknown activity, reinforcing the idea that the functional characterization of the scorpion venoms is far from exhausted.

Although we focused on studies describing the New-World venomics, it is important to highlight that a number of venomic studies from Old-World scorpions have also been published [91,95-101]. 

For example, Xu et al. [100] used a proteomic strategy that combined multidimensional protein separation techniques (2-DE, SDS-PAGE, and RP-HPLC) with MS (ESI-Q-TOF MS and MALDI-TOF MS) to analyze the venom proteome of *Mesobuthus martensii*. The authors reported 227 peptides or proteins unambiguously identified, 115 of which were confirmed at the protein level from the crude venom, including 24 typical toxins, 7 atypical toxins, 12 venom enzymes, and 72 cell-associated proteins. Noteworthy, seven novel toxins belonging to typical toxins were also found in the *M. martensii* venom.

Ma et al. [101] used a combination of expressed sequence tag (EST)-sequencing data from transcriptome analysis and MS-based proteomic methods on *Heterometrus petersii* venom. In total, 10 known and 12 unknown atypical toxin types, and 184 non-redundant venom toxins were identified. The diversity of the venome was demonstrated by the presence of at least 22 venom peptide families. Concurrently, numerous venom peptide families showed high homology with toxins from other animal species, indicating compositional convergence. 

PTMs significantly change the physicochemical properties (e.g., structure, affinity, stability, interaction, etc.) of proteins, and so to animal protein toxins too. PTMs are frequently studied in cone snails’, wasps’ and snakes’ venoms but somehow are overlooked in scorpions’ and spiders’ venomic papers. To the best of our knowledge, there is only one proteomic paper reporting that *Tityus serrulatus* venom proteins and peptides are subjected to PTMs (i.e., phosphorylation, *N*-linked glycosylation, and proteolysis) [82]. Despite the lack of such studies, PTMs do play an important role in the activity of arachnid venom proteins, as shown by Veiga et al. [102]. The enzymatic removal of potential N-glycosylated proteins in the venom of *Loxosceles intermedia* reduced the dermonecrotic and gelatinolytic activities of the crude venom. Thus, we urge our scientific community to pursue such task, employing enrichment steps for PTMs (phosphorylation, glycosylation, acetylation, etc.) on proteomic workflows, as illustrated in Figure 2, to shed more lights on the molecular complexity of scorpions’ and spiders’ venoms.

Finally, Table 1 summarizes key information related to the venomic studies cited in this review, including animal (spider or scorpion) genera, proteomic method and MS platform employed. Figure 3 represents such information as an interaction map. The nodes represent the terms reported in the table (arachnid genera, MS platform and proteomic strategy, also including the papers’ corresponding authors). Node size represents the number of publications in which each term appeared. It is possible to observe in the Figure 3 that PTMs are indeed overlooked in venom proteome studies of scorpions and spiders.

**Table 1.** List of publications on spider and scorpion venomics cited in this review.

Ref.	Arachnid	Genera	Transcriptome	Proteomic method	MS platform
[7]	Scorpion	*Tityus*	Yes	Shotgun/Bottom-up	ESI-Orbitrap
[35]	Spider	*Phoneutria*	No	2-DE	ESI-Q-TOF MALDI-TOF
[40]	Scorpion	*Tityus*	No	PMF	MALDI-TOF ESI-QQQ
[46]	Scorpion	*Tityus*	No	Shotgun/Bottom-up	ESI-Orbitrap
[57]	Spider	*Loxosceles*	No	2-DE	ESI-Q-TOF MALDI-TOF ESI-QQQ
[58]	Spider	*Ornithoctonus*	No	2-DE	ESI-Q-TOF MALDI-TOF
[60]	Spider	*Heteropoda Poecilotheria Viridasius* *Latrodectus*	Yes	Top-down/Peptidome	ESI-Orbitrap
[61]	Spider	*Cupiennius*	Yes	Shotgun/Bottom-up Top-down/Peptidome	ESI-Orbitrap
[62]	Spider	*Phoneutria*	Yes	Shotgun/Bottom-up	ESI-Orbitrap
[63]	Spider	*Phlogius*	No	Shotgun/Bottom-up Top-down/Peptidome	ESI-Q-TOF
[64]	Spider	*Acanthoscurria*	Yes	Shotgun/Bottom-up Top-down/Peptidome	ESI-Q-TOF ESI-Orbitrap
[65]	Spider	*Stegodyphus* *Acanthoscurria*	Yes	Shotgun/Bottom-up	ESI-Q-TOF
[66]	Spider	*Physocyclus*	Yes	Shotgun/Bottom-up	ESI-Orbitrap
[67]	Spider	*Ornithoctonus*	Yes	Top-down/Peptidome Shotgun/Bottom-up	MALDI-TOF
[68]	Spider	*Hadronyche*	Yes	PMF Shotgun/Bottom-up	MALDI-TOF ESI-Q-TOF ESI-Orbitrap
[69]	Spider	*Tetragnatha*	Yes	Shotgun/Bottom-up	ESI-Orbitrap
[70]	Spider	*Loxosceles*	No	Shotgun/Bottom-up Top-down/Peptidome	ESI-Orbitrap
[71]	Spider	*Grammostola*	No	Shotgun/Bottom-up	ESI-Orbitrap
[72]	Spider	*Phoneutria*	No	Shotgun/Bottom-up	ESI-Orbitrap ESI-Q-TOF
[73]	Spider	*Scytodes*	Yes	Shotgun/Bottom-up	ESI-Orbitrap
[74]	Scorpion	*Tityus*	No	PMF	MALDI-TOF ESI-IT
[75]	Scorpion	*Tityus*	No	PMF	MALDI-TOF
[76]	Scorpion	*Rhopalurus*	No	PMF	ESI-IT
[77]	Scorpion	*Rhopalurus*	No	PMF	ESI-Orbitrap
[78]	Scorpion	*Tityus* *Leiurus*	No	PMF	ESI-Q-TOF
[79]	Scorpion	*Tityus*	No	PMF	ESI-IT MALDI-TOF
[80]	Scorpion	*Tityus*	No	PMF Shotgun/Bottom-up	ESI-IT
[81]	Scorpion	*Tityus*	No	Shotgun/Bottom-up	ESI-Orbitrap
[82]	Scorpion	*Tityus*	No	Shotgun/Bottom-up Top-down/Peptidome PTMs	ESI-Orbitrap
[83]	Scorpion	*Tityus*	Yes	Shotgun/Bottom-up	ESI-Orbitrap
[84]	Scorpion	*Tityus*	No	PMF/Shotgun/Bottom-up	ESI-IT ESI-Orbitrap
[85]	Scorpion	*Tityus*	No	Top-down/Peptidome	ESI-IT-TOF
[86]	Scorpion	*Tityus*	No	PMF	ESI-IT
[87]	Scorpion	*Rhopalurus*	No	Shotgun/Bottom-up	ESI-Orbitrap
[88]	Scorpion	*Centruroides*	Yes	Shotgun/Bottom-up	ESI-Orbitrap
[89]	Scorpion	*Centruroides*	Yes	Shotgun/Bottom-up	MALDI-TOF
[90]	Scorpion	*Centruroides*	Yes	PMF Shotgun/Bottom-up	ESI-Orbitrap
[91]	Scorpion	*Centruroides*	Yes	Shotgun/Bottom-up	ESI-Orbitrap
[92]	Scorpion	*Centruroides*	No	2-DE	ESI-Orbitrap
[93]	Scorpion	*Centruroides*	Yes	PMF	ESI-IT ESI-Orbitrap
[95]	Scorpion	*Buthus*	No	Shotgun/Bottom-up Top-down/Peptidome	ESI-Orbitrap
[96]	Scorpion	*Heterometrus*	No	Shotgun/Bottom-up	MALDI-TOF
[97]	Scorpion	*Androctonus*	No	PMF	MALDI-TOF
[98]	Scorpion	*Heterometrus*	Yes	2-DE	ESI-Q-TOF MALDI-TOF
[99]	Scorpion	*Mesobuthus*	Yes	Shotgun/Bottom-up	ESI-Q-TOF
[100]	Scorpion	*Mesobuthus*	No	PMF 2-DE	MALDI-TOF ESI-Q-TOF
[101]	Scorpion	*Heterometrus*	Yes	Shotgun/Bottom-up	ESI-Q-TOF

## Conclusion

We presented in this review historical landmarks of venomic studies on scorpion and spider venoms. It is fascinating to observe how venomics has evolved as MS instrumentation and proteomic methods have improved. From descriptive papers relying on PMF, we now find comprehensive venom characterization by means of omics methods. We prepared a table presenting the most important information from studies on venomics covered in this review article, including instrumentation and methods employed. 

### Abbreviations

1-DE: one-dimensional gel electrophoresis; 2-DE: two-dimensional gel electrophoresis; CIEX: cation exchange chromatography; ESI: electrospray ionization; HPLC: high-pressure liquid chromatography; IT: ion trap; LC: liquid chromatography; MALDI: matrix-assisted laser desorption/ionization; MS/MS: tandem mass spectrometry; MS: mass spectrometry; NMR: nuclear magnetic resonance; PMF: peptide mass fingerprinting; Q: quadrupole; QQQ: triple quadrupole; RP: reverse-phase; TOF: time-of-flight.
